# Supplementary material for: Dietary and Genetic Cholesterol Loading Rather Than Steatosis Promotes Liver Tumorigenesis and NASH-Driven HCC
Source: Cancers (Basel). 2021 Aug 13;13(16):4091. doi: 10.3390/cancers13164091 (PMC8393403; doi:10.3390/cancers13164091)
Supplement: Supplementary file 1 [file cancers-13-04091-s001.zip › cancers-1267241-supplementary.pdf]

Supplementary Figures and Tables

Figure S1. MUP-uPA liver regeneration in a regular, low fat diet.

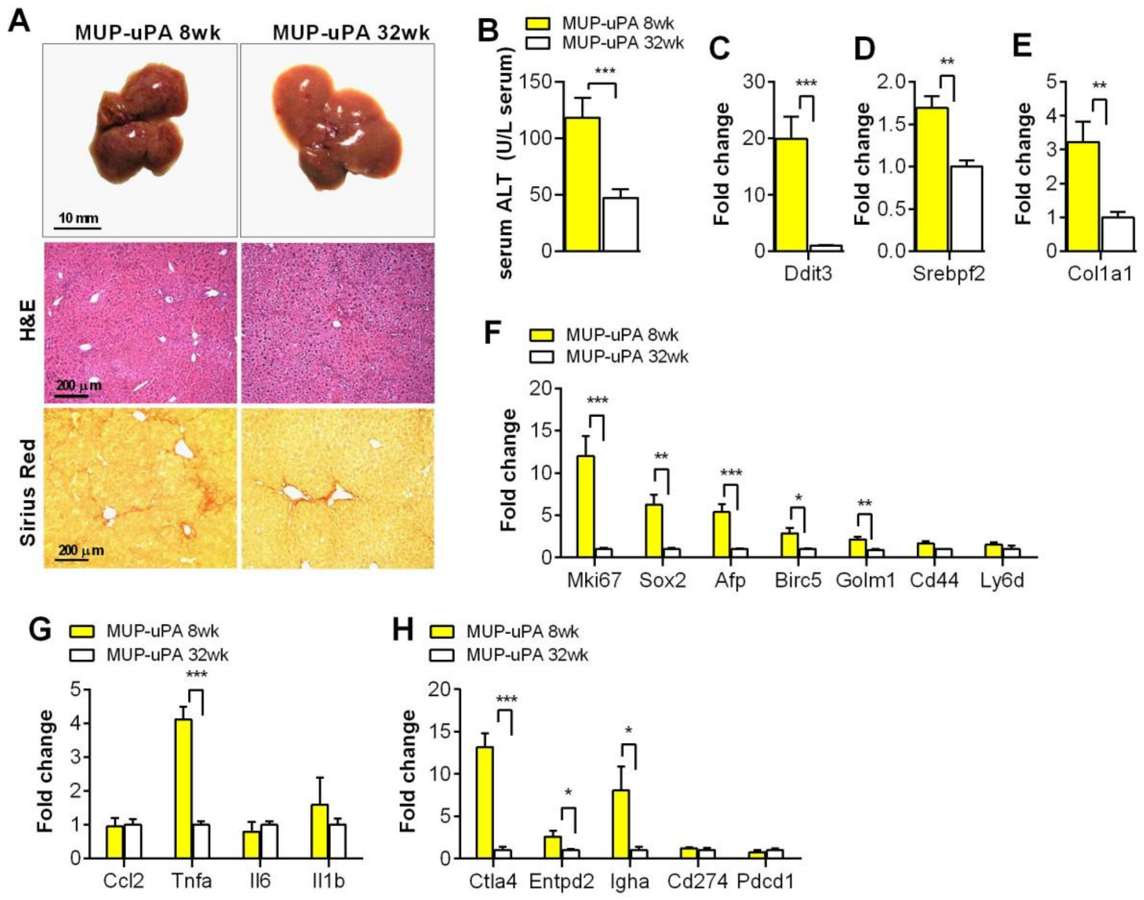

Figure S1. Comparison of 8-weeks age animals to 32 weeks of age MUP-uPA transgenic animals kept in regular diet (RD) which restored liver tissue and functionality. A) Representative whole liver images, histological staining for hematoxylin-eosin (H&E), and collagen fibers (Sirius red) of paraffin-embedded liver sections. B) Transaminase serum levels (ALT). C) Transcript qPCR quantification of the ER stress regulated gene Chop (Ddit3). D) Expression of the mRNA of cholesterol metabolism master regulator SREBP2 (Srebpf2). E) Transcript expression of collagen 1 (Col1a1). F) Messenger RNA expression of proliferation-related gene (Ki67), hepatocyte stemness gene (Sox2), and several HCC-associated genes (Afp, Birc5, Golm1, Cd44 and Ly6d). G) Expression of mRNA of inflammatory genes, Mcp-1 (Ccl2), tumor necrosis-alpha (Tnfα), interleukin 6 (Il6), Interleukin 1 beta (Il1b). H) Expression of immune checkpoint and tolerance-related genes, Ctla4, Entpd2, Immunoglobulin A isotype (Igha), Pd-1 (Cd274), and pd-1 ligand (Pdcd1). (8wks group n= 5, 32wks group n=6 animals). Data from qPCR corrected by a housekeeping gene (Gapdh) and relative to controls (32wk group). All values are mean ± SEM, symbols *, ** or *** indicate statistically significant differences ($p<0.05$, $p<0.01$, $p<0.001$ respectively) on Student's t-test.

Figure S2. Cholesterol supplementation enhances NASH-associated liver tumorigenesis in MUP-uPA mice.

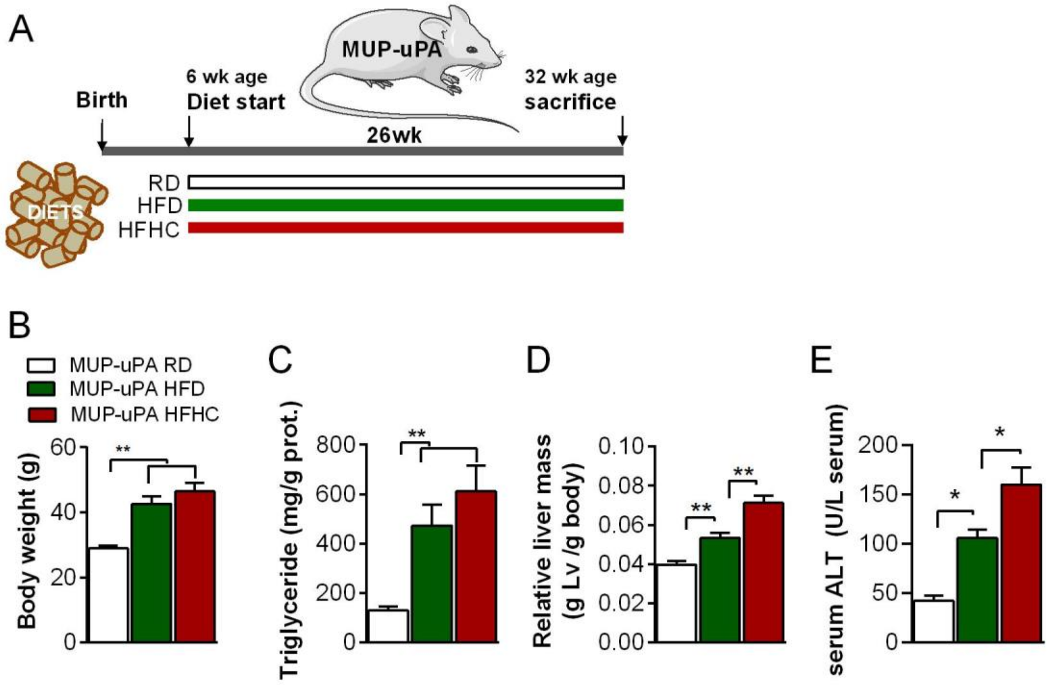

Figure S2. A) Schematic illustration of the experimental design in which mice were fed a regular diet (RD), a high fat diet (HFD), or cholesterol-supplemented HFD (HFHC) diet for 26 weeks. N per group: RD (12), HFD (12), HFHC (17). B) Animal body weight after 26 weeks on the specified diets. C) Liver tissue triglyceride composition from homogenates. D) Relative liver to body weight ratio. E) Transaminase serum levels (ALT). All values are mean ± SEM, symbols *, ** or *** indicate statistically significant differences ($p<0.05$, $p<0.01$, $p<0.001$ respectively) on One-way ANOVA and Bonferroni post-test.

Figure S3. Cholesterol supplementation enhances tumorigenesis in the liver induced by DEN plus HFD.

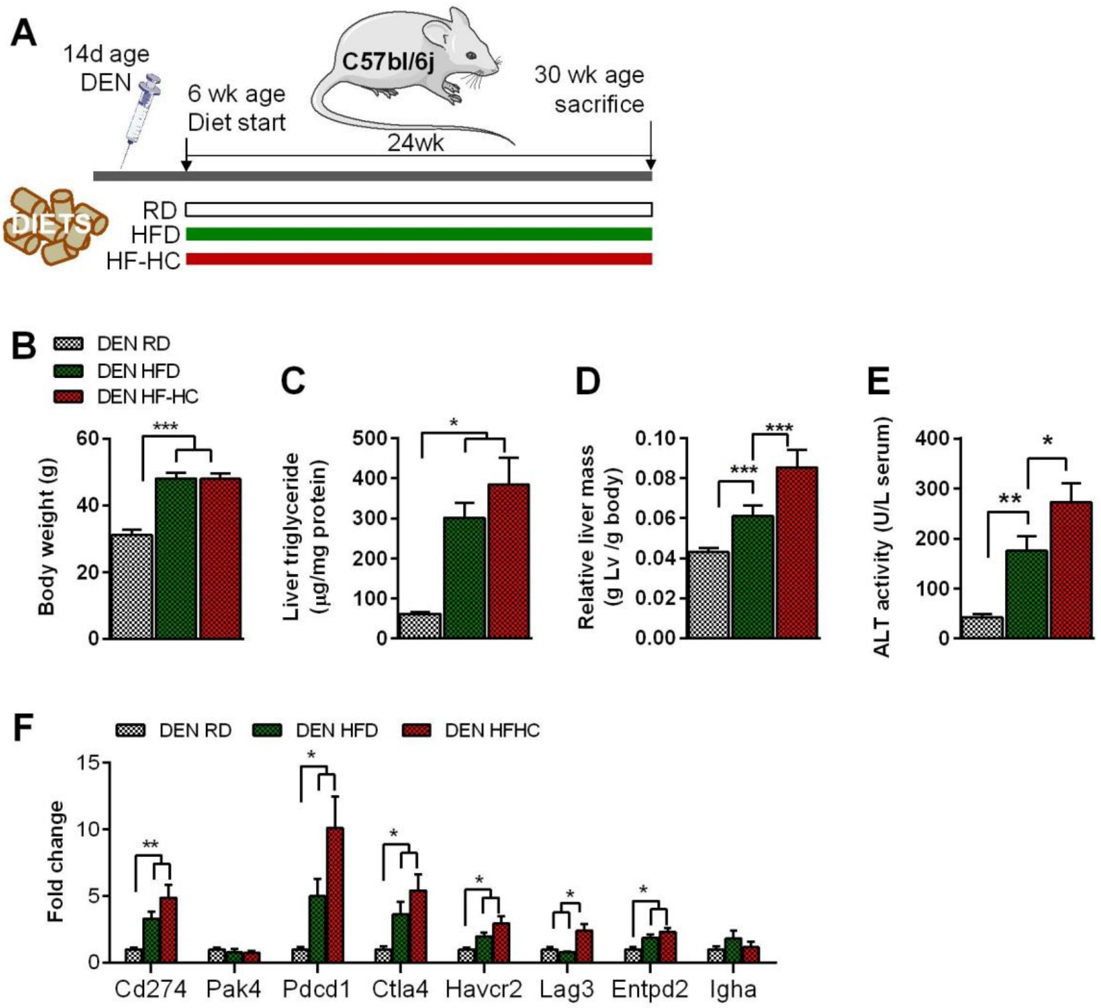

Figure S3. A) Schematic illustration of the experimental design, by treating acutely with a single DEN injection to the animals at 14 days of age and continuing the feeding with regular diet (RD), or changing at 6 weeks of age to high fat diet (HFD), or cholesterol-supplemented HFD (HFHC) diet for 24 weeks. N per group: RD (7), HFD (11), HFHC (11). B) Animal body weight after 24 weeks on the specified diets. C) Liver tissue triglyceride composition from homogenates. D) Relative liver to body weight ratio. E) Transaminase serum levels (ALT). H) Liver mRNA expression of immune checkpoint and immune suppression genes Pd-1L (Cd274), Pd-1 (Pdcd1), Ctla-4, Havcr2, Lag3, Entpd2). N=6 per group. All values are mean ± SEM, symbols *, ** or *** indicate statistically significant differences ($p<0.05$, $p<0.01$, $p<0.001$ respectively) on One-way ANOVA and Bonferroni post-test.

Figure S4. Uncropped immunoblots from Western Blot panels.

Figure 1F and 1H

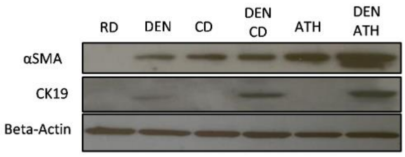

Original films

Rat αSMA (42kDa)

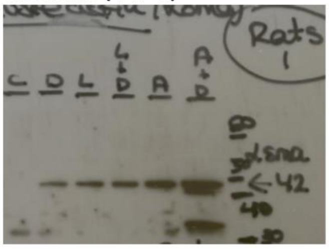

Rat CK19 (40kDa)

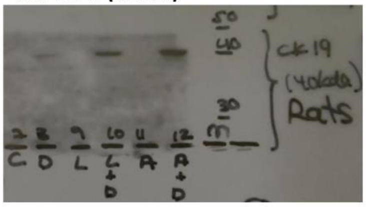

Rat β actin (42kDa)

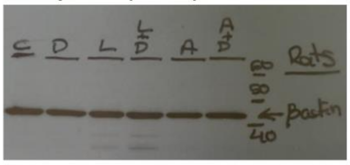

Figure 2F and 2I

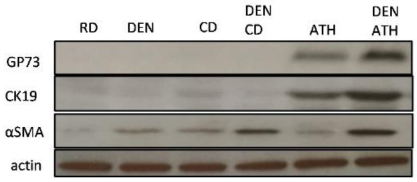

Original films

Mouse αSMA (42kDa)

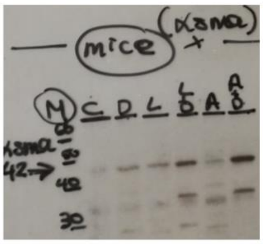

Mouse CK19 (40kDa)

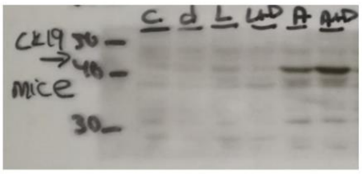

Mouse β-actin (42kDa)

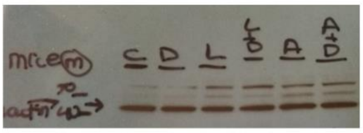

Mouse Gp73 (73kDa)

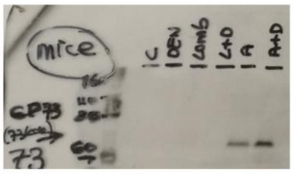

Figure 3H

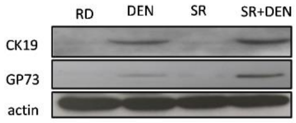

Original films

Mouse CK19 (40kDa)

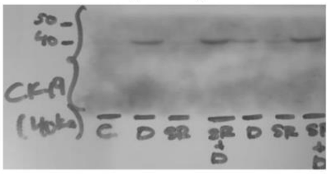

Mouse Gp73 (73kDa)

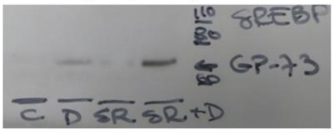

Mouse β-actin (42kDa)

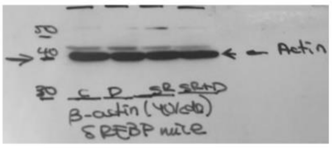

Table S1. Animal models of NASH-derived HCC used in this study.

	Animal	HCC inducer	Diets	Time on treatment
figure 1	Sprague-Dawley Rats	None (Ctrl)	Regular diet (RD)	10 Weeks
			Colin deficient (CD)	
			Atherogenic diet (ATH)	
		DEN in drinking water	Regular diet (RD)	
			Colin deficient (CD)	
			Atherogenic diet (ATH)	
figure 2	C57bl6/j mice	None (Ctrl)	Regular diet (RD)	20 Weeks
			Colin deficient (CD)	
			Atherogenic diet (ATH)	
		DEN ip. at day 14	Regular diet (RD)	
			Colin deficient (CD)	
			Atherogenic diet (ATH)	
figure 3	C57bl6/j mice	None (Ctrl)	Regular diet (RD)	24 Weeks
		DEN in drinking water		
	SREBP-2 Tg mice	None (Ctrl)		
		DEN in drinking water		
figure 4	C57bl6/j mice	None	Regular diet (RD)	40 Weeks
			High Fat diet (HFD)	
			High Fat High Cholesterol (HFHC)	
figure 5	MuP-uPA	None	Regular diet (RD)	26 Weeks
			High Fat diet (HFD)	
			High Fat High Cholesterol (HFHC)	
figure 6	C57bl6/j mice	DEN ip. at day 14	High Fat High Cholesterol (HFHC)	24 Weeks
			HFHC plus Ezetimibe (EZE)	
	MuP-uPA	None	High Fat High Cholesterol (HFHC)	26 Weeks
			HFHC plus Ezetimibe (EZE)	

Table S2. Oligonucleotide sequences used for qPCR.

Specie	GeneSymbol	NCBI Ref.Seq	Forward	Reverse
Mm	*Acta2*	NM_007392	GTTCAGTGGTGCCTCTGTCA	GGGATCCTGACGCTGAAGTA
Mm	*Actb*	NM_007393	GACGGCCAGGTCATCACTAT	CGGATGTCAACGTCACACTT
Mm	*Afp*	NM_007423	CAGCAGCCTGAGAGTCCATA	GGCGATGGGTGTTTAGAAAG
Mm	*Atf4*	NM_009716	ATGACCGAAATGAGCTTCCTG	CTG TCC CGG AAA AGG CAT CC
Mm	*Birc5*	NM_009689	TCTTGACAGTGAGGAAGGCG	TACCGAGAACGAGCCTGATT
Mm	*Ccl2*	NM_011333	GCTGCTGGTGATCCTCTTG	CAGCCAGATGCAGTTAACGC
Mm	*Ccne1*	NM_007633	GGGATTTCAGCCTTATTTATTGC	GCCACTTAAGGGCCTTCATC
Mm	*Cd274*	NM_021893	CCTCGCCTGCAGATAGTTCC	GACGTTGCTGCCATACTCCA
Mm	*Cd44*	NM_009851	GTCCGGGAGATACTGTAGCG	CAAGTTTTGGTGGCACACAG
Mm	*Col1a1*	NM_007742	GAGCGGAGAGTACTGGATCG	GTTCGGGCTGATGTACCAGT
Mm	*Ctla4*	NM_001281976	ATGGCTTGTCTTGGACTCCG	TTGGGTCACCTGTATGGCTTC
Mm	*Ddit3*	NM_007837	CCA CCA CAC CTG AAA GCA GAA	AGG TGA AAG GCA GGG ACT CA
Mm	*Entpd2*	NM_009849	CGGCCTCCTGCTACTGTG	GGCCACTTGTAGACAAACATGG
Mm	*Gapdh*	NM_008084	TTGAGGTCAATGAAGGGGTC	TCGTCCCGTAGACAAAATGG
Mm	*Golm1*	NM_027307	CAGCTGGAGAATGTCAACAAGC	TGCTGTAACTCCTCTGCAGTG
Mm	*Gpc3*	NM_016697	CGTTGGTGTAGTTCTTGGCA	CAACTAACAGCACGGCTGAA
Mm	*Havcr2*	NM_134250	ACCCTAACCACGGAGAGAAATG	GCAGTTCTGATCGTTTCTCCAG
Mm	*Hmgcr*	NM_008255	CACCTCTCCGTGGGTTAAAA	GAAGAAGTAGGCCCCCAATC
Mm	*Igha*	NC_000078	CGCATCATTCAAGTGCACAG	GCTCATTCAGGGCCAGCTC
Mm	*Il1b*	NM_008361	GAGCTGAAAGCTCTCCACCTC	CTTTCCTTTGAGGCCCAAGGC
Mm	*IL6*	NM_031168.1	AGTCCGGAGAGGAGACTTCA	TTGCCATTGCACAACTCTTT
Mm	*Krt19*	NM_008471	GTGAAGATCCGCGACTGG	GACAATCTTGGAGTTGTCAATGG
Mm	*Lag3*	NM_008479	TCAATGCCACTGTCACGTTG	TTTCCAGATGCCGGGGTTAC
Mm	*Ly6d*	NM_010742	CTCCACTGAGGTGACGGTTT	TCTGCTCGTCCTCCTTGTCT
Mm	*Lyve1*	NM_053247	GTCCAACACGGGGTAAAATG	AGAATGGCAAAGGTGTCCTG
Mm	*Mki67*	NM_001081117	ATCATTGACCGCTCCTTTAGGT	GCTCGCCTTGATGGTTCCT
Mm	*Pak4*	NM_027470	AATCAGCACGCAGACCCAAG	TCAAGCAGCAGAGTGAGAGC
Mm	*Pcna*	NM_011045	ATGCCGTCGGGTGAATTTG	TCTCCAATGTGGCTAAGGTCTC
Mm	*Pdcd1*	NM_008798	CGGTTTCAAGGCATGGTCATT	CCTCCTTCAGAGTGTCGTCC
Mm	*Pdia4*	NM_011032	CAA GAT CAA GCC CCA CCT GAT	AGT TCG CCC CAA CCA GTA CTT
Mm	*Sox2*	NM_011443	AGTACAACTCCATGACCAGCTC	ACTTGACCACAGAGCCCATG
Mm	*Spp1*	NM_009263	TCTGCTTCTGAGATGGGTCA	TTGGCAGTGATTTGCTTTTG
Mm	*Srebpf2*	NM_033218.1	GCGCCAGGAGAACATGGT	CGATGCCCTTCAGGAGCTT
Mm	*Tgfβ1*	NM_011577	TCAGACATTCGGGAAGCAG	CAAGGTAACGCCAGGAATTG
Mm	*Tnfα*	NM_013693.2	CCAGACCCTCACACTCAGATC	CACTTGGTGGTTTGCTACGAC
Rn	*Acta2*	XM_032891814	GATCACCATCGGGAATGAACGC	CTTAGAAGCATTTGCGGTGGAC
Rn	*Afp*	NM_012493	CAGTGAGGAGAAACGGTCGG	ATGGTCTGTAGGGCTCGGCC
Rn	*Col1a1*	NM_053356	CAATCCATCCATCCAGACCGTTGTG	CCTCAAGGTTTCCAAGGACC
Rn	*Hif1α*	NM_024359	GCCCAGTGAGAAAGGGGAAA	CATGAATGTGGCCTGTGCAG
Rn	*Krt19*	NM_199498	CACTTGTACCCTGGCAACCA	GTGTCAGCACGCACGTTACT
Rn	*Star*	NM_000349	TTAATCCACGTGCTAGGGGT	CCTACAGACACATGCGCAAC
Rn	*Stard3*	NM_006804	AGTGAGGAGCCCAGGGAG	CCGTGGCTGACATGGAG
Rn	*Tgfβ1*	NM_021578	ACCGCAACAACGCAATCTAT	GTAACGCCAGGAATTGTTGC
Rn	*Vegfα*	NM_001110335	GGTGCTACCTAGTGGGTGGA	CTGGCCTTAAGGAGCACTTG
